# S-1 versus S-1 plus cisplatin concurrent intensity modulated radiation therapy in the treatment of esophageal squamous cell carcinoma: Study protocol for a randomized controlled phase II trial

**DOI:** 10.1097/MD.0000000000008998

**Published:** 2017-12-08

**Authors:** Yixue Wen, Zhenhuan Zhao, Jidong Miao, Qilin Yang, Yan Gui, Mingqiang Sun, Honggang Tian, Qiang Jia, Dongbiao Liao, Chen Yang, Xiaobo Du

**Affiliations:** a Department of Oncology, Mianyang Central Hospital, Mian Yang; b Department of Oncology, Affiliated Hospital of North Sichuan Medical College, Nan Chong; c Department of Oncology, Zigong Fourth People's Hospital, Zi Gong; d Department of Oncology, Ziyang People's Hospital, Zi Yang; e Department of Oncology, Guangyuan First People's Hospital, Guang Yuan; f Department of Oncology, Jiangyou People's Hospital; g Department of Oncology, Jiangyou Second People's Hospital, Jiang You; h Department of Oncology, Jianyang People's Hospital, Jian Yang, Sichuan, People's Republic of China.

**Keywords:** cisplatin, concurrent chemoradiotherapy, esophageal squamous cell carcinoma, S-1

## Abstract

**Introduction:**

Chemotherapy regimens are often a 2-drug regimen in concurrent chemotherapy and radiotherapy for esophageal cancer (EC). However, some retrospective studies have suggested that for patients with EC receiving radiotherapy combined with 2-drug chemotherapy have the severe toxicity. And S-1 alone with the combination of radiotherapy treatment effect is good, and achieved good clinical remission rate. The purpose of this trial is compare the efficacy and toxicity of combining S-1 or S-1 plus cisplatin with radiotherapy for esophageal squamous cell carcinoma.

**Methods/Design:**

The study is a randomized, controlled, multicenter trial, comparing S-1 versus S-1 plus cisplatin concurrent radiotherapy for patients with esophageal squamous cell carcinoma. Eighty-eight patients with unresectable or medically unfit for surgery esophageal squamous cell carcinoma (clinical stage I to III), will randomly assigned to receive four cycles (2 concomitant and 2 postradiotherapy) S-1 or S-1 plus cisplatin along with radiotherapy 60–66 Gy/30 to 33 fractions. The primary outcome is complete response rate of primary tumor which will be measured by endoscopy and computer screen at 3 months after the completion of treatment. Secondary outcomes include survival and toxicity.

**Discussion:**

To our knowledge, this study protocol is the first to test the effect between S-1 versus S-1 plus cisplatin concurrent intensity modulated radiation therapy in the treatment of esophageal squamous cell carcinoma. If the result will be the same effect and fewer side effects and less costly in S-1 plus radiotherapy. It will supply more treatment selection for esophageal squamous cell carcinoma.

## 1 Introduction

Esophageal cancer (EC) is a common malignancy worldwide.^[1]^ Squamous cell carcinoma is the most common histological type of EC worldwide, with a higher incidence in developing nations.^[2]^ In China, in 2015, the total incidence and mortality rates of EC were 477.9 and 375.0 per 100,000 persons, respectively, and over 90% of cases are esophageal squamous cell carcinoma.^[3,4]^ The incidence and mortality of EC ranks third in China. Yanting in Mianyang city is one of the highest risk areas for EC in the world.^[5]^

Surgery, radiation therapy, and chemotherapy are the major treatments for EC. But at initial diagnosis, 40% to 60% of patients are not candidates for surgery.^[6]^ In these patients, definitive chemoradiotherapy (CRT) is the established treatment of choice. Early trials have shown beneficial effects of CRT compared to radiotherapy alone,^[7,8]^ and definite CRT has been shown in smaller studies to be comparable in efficacy to surgery in patients with nonmetastatic disease.^[9,10]^ Concomitant use of chemotherapy and a radiation dose schedule that is more efficient compared to conventional radiotherapy may provide better outcomes in patients with EC.^[11]^ The chemotherapeutic drugs cisplatin and 5-fluorouracil have been most commonly used in studies examining the effects of definitive CRT in EC.^[12]^ However, despite many advances in diagnosis and treatment, the 5-year survival rate for patients diagnosed with EC ranges from 15% to 20%.^[12]^

Chemotherapy regimens are often a 2-drug regimen in concurrent chemotherapy and radiotherapy for EC.^[13]^ However, some retrospective studies have suggested that for patients with EC receiving radiotherapy combined with 5-FU/cisplatin chemotherapy, due to severe toxicity, only 9% to 38.5% of patients completed the planned treatment. In addition, one study showed that patients receiving radiotherapy combined with 5-FU/CDDP chemotherapy experienced a treatment-related mortality as high as 18%.^[7,14–17]^ Therefore, it is necessary to explore the toxicity of the combined program to improve its treatment tolerance and safety.

In recent years, S-1 reflects the good effect, single use or combined with platinum or combined with radiotherapy have achieved good results, especially the S-1 alone with the combination of radiotherapy treatment effect is very good, and achieved good clinical remission rate (CR).^[18–22]^

Therefore, we designed a prospective, randomized, multicenter phase II trial to compare the efficacy and toxicity of combining S-1 or S-1 plus cisplatin with intensity modulated radiation therapy for esophageal squamous cell carcinoma.

## 2 Objectives

### 2.1 Primary

The primary outcome is the rate of complete response.

### 2.2 Secondary

The secondary outcomes are the overall survival rate, progression-free survival rate, toxicity.

## 3 Methods/design

### 3.1 Recruitment and study design

**Figure 1:**
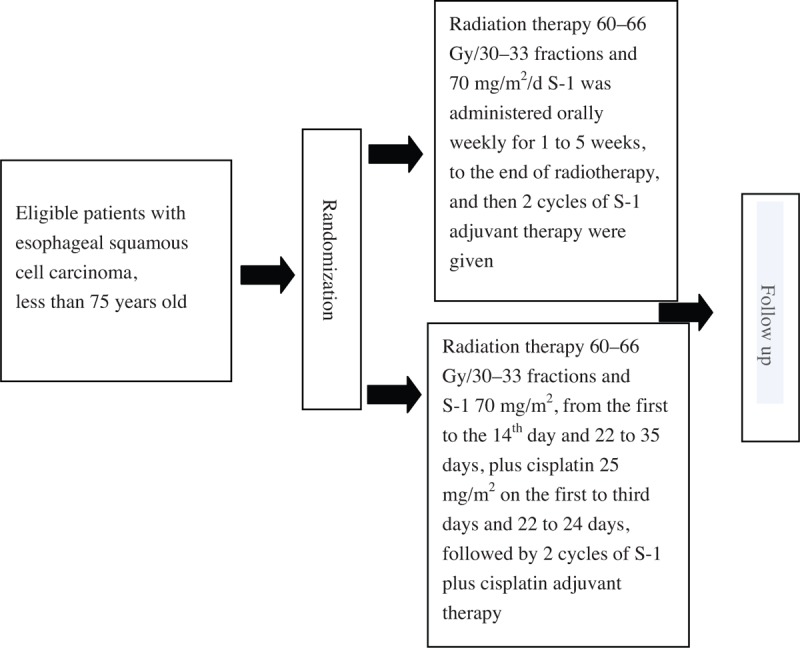
The plan of the clinical trial.

This trial is a prospective, randomized, multicenter phase II clinical trial. Patients with unresectable or medically unfit for surgery and no distant metastasis of esophageal squamous cell carcinoma will be randomized into 2 groups (Fig. 1): one group receiving S-1 concurrent intensity-modulated radiotherapy treatment (experimental group) and S-1 plus cisplatin concurrent intensity modulated radiation therapy (control group).

Before the start of treatment, the recruitment of eligible participants firstly includes the explanation, to each potential study participant, of the common ethical issues, purpose, design, description of the 2 different chemotherapy regimens, implementation, requirements, timeline of this study.

#### 3.1.1 Inclusion criteria

1. Histology or cytology confirmed esophageal squamous cell carcinoma.
2. There are measurable lesions according to the Response Evaluation Criteria in Solid Tumors (RECIST) standard.
3. Patients with technically unresectable cancer or those with surgical contraindications and those who refused to undergo surgery.
4. Clinical stage I to III.
5. Age 18 to 75.
6. ECOG physical performance score 0 to 1.
7. No esophageal perforation or active esophageal bleeding, no obvious trachea or thoracic major vascular invasion.
8. Chest radiotherapy, chemotherapy, immunotherapy, or biological therapy has not been performed before.
9. Hemoglobin is greater than 100 g/L, platelet over 100 × 10^9^/L, absolute neutrophil count over 1.5 × 10^9^/L.
10. The serum bilirubin is less than or equal to 1.5 times the upper normal limit (UNL).
11. AST (SGOT) and ALT (SGPT) are less than or equal to 2.5 times the UNL.
12. Alkaline phosphatase is less than or equal to 5 times the UNL.
13. Serum creatinine or creatinine clearance is less than or equal to 1.25 times the UNL.
14. Without interstitial pneumonia or a history of interstitial pneumonia.
15. Have signed a Declaration of Informed Consent.

#### 3.1.2 Exclusion criteria

1. A history of chest radiotherapy, chemotherapy, or surgical resection of EC.
2. The primary lesions are multifocal EC.
3. The distance between the gastroesophageal junction and the lower bound of the esophageal primary lesion is less than 3 cm.
4. Severe cardiovascular or pulmonary disease, interstitial pneumonia or a history of interstitial pneumonia.
5. Patients with distant metastases.
6. There are obvious esophageal ulcers, moderate pain in the chest and back, or symptoms of esophageal perforation.
7. Patient cannot understand the test requirements, or patients may not be able to comply with the requirements of the tests.
8. There are other malignant lesions in the patient, not including curable skin cancer (non melanoma), cervical carcinoma in situ, or malignant disease cured ≥5 years prior.
9. Known to have 3 to 4 levels of allergic reactions to the treatment.
10. Participated in other clinical trials in the past 30 days.
11. Patient's esophagus is completely blocked or cannot swallow the S-1.
12. Patients have esophageal stent.
13. The researchers believe that there is an obvious disease present, meaning that the patient should be excluded from this study.

#### 3.1.3 Drop-out criteria

Criteria that will lead to the drop-out of a patient before completion of this study may be any kind of treatment for medical reasons or impairment that will result in an interruption or early completion of treatment, include:

1. The withdrawal of the informed consent of patients.
2. Patient safety events.
3. Chemotherapy delays because of toxicity lasting for more than 2 weeks.
4. The patient enrolled in another study during the clinical study.
5. Patient does not want to continue to participate.
6. The compliance of the patient is not sufficient.
7. The researchers’ judgment.

### 3.2 Radiotherapy

All patients receive a planning computed tomography (CT) scan with a slice thickness of 3 to 5 mm. Afterwards these CT scans are used to outline organs at risk (OARs) and to contour the gross tumor volume (GTV) and clinical target volume (CTV). The GTV was contoured based on the esophageal endoscopy, barium esophagography, and chest CT scans. The clinical target volume (CTV) consisted of the GTV plus a 0.5 to 1 cm circumferential margin, 3 to 4 cm craniocaudal margin and lymph node drainage area. The supraclavicular nodes were included for upper esophageal lesions, and celiac nodes were included for distal esophageal lesions. The planning gross tumor volume (PGTV) consisted of the GTV plus a 0.5 to 1 cm circumferential margin, 3 cm craniocaudal margin. The planning target volume (PTV) consisted of the CTV plus a 0.5 to 1 cm margin for daily set-up error and organ motion. Radiotherapy will be performed as intensity modulated radiotherapy (IMRT), which will be administered beginning on day 1 of chemotherapy using a linear accelerator (energy >6 MV) in 30 to 33 fractions (PTV54–59.4 Gy/30–33 fractions, 1.8 Gy/time dose fractionation; PGTV 60–66 Gy/30–33 fractions, 2 Gy/time dose fractionation, 5 fractions per week [Monday to Friday], one fraction per day).

### 3.3 Chemotherapy

The experimental group will receive chemotherapy S-1 70 mg/m^2^ weekly from Monday to Friday until radiotherapy completion, then 2 weeks after completion of radiotherapy, 2 cycles of adjuvant chemotherapy consisting of S-1 70 mg/m^2^ from 1 to 14 days will be administered, each cycle consisting of 21 days. The control group will receive chemotherapy S-1 70 mg/m^2^ for the first 14 and 22 to 35 days, plus cisplatin 25 mg/m^2^ for the first 3 and 22 to 24 days. Two weeks after the end of radiotherapy, 2 cycles of adjuvant chemotherapy consisting of cisplatin 25 mg/m^2^ for the first 3 days and S-1 70 mg/m^2^ from 1 to 14 days will be administered, each cycle consisting of 21 days.

### 3.4 Data collection and management

Each case will be completed with a case report. Mianyang Central Hospital is responsible for establishing the database and entering the data, and will use a date acquisition system electronic data management system to manage the data. The main researchers and statistics analyzer will audit the data and lock it. The final version of the statistical plan will be completed and used to analyze the locked data.

*Special data processing*: Principles for evaluation of the effectiveness of the objective. When the month and year are clear, but the day is unknown, the date will be recorded as the fifteenth day of the month. When the year is clear, but the day and month are unknown, the date will be recorded as an intermediate point between the end of the year and the last known date.

### 3.5 Assessment of the primary and secondary endpoints

In this trial, endoscopic complete response (CR) 3 months after completion of treatment will be evaluated as the primary endpoint. Endoscopic CR was defined as the complete disappearance of any tumor ulceration or stenosis with no new lesion (all endoscopic pictures and reports had to be available), observation of the entire esophagus as defined by Tahara et al,^[22]^ but also no CT-scan progression. Biopsies were not mandatory. Tumor responses were assessed during week 15 according to RECIST guidelines.^[23]^ The primary tumor was assessed on CT scan with measure of the vertical length and maximal thickness of the esophageal wall on transverse plane. Secondary endpoints are the toxicity, progression-free survival (PFS), and overall survival (OS). Adverse events will be encoded in accordance with the requirements of Med DRA. Severity of adverse events will be graded according to the NCI-CTC classification criteria. The toxicity of the treatment, including acute and chronic toxicity will continue to be evaluated during treatment and during follow-up (Table 1). Any serious adverse drug reactions will be reported promptly to the hospital ethics committee. An intent-to-treat analysis (on all randomized patients) was planned for all other end points. PFS was defined as the date from randomization to tumor progression or death. OS was measured from date of study entry to date of death.

**Table 1:**
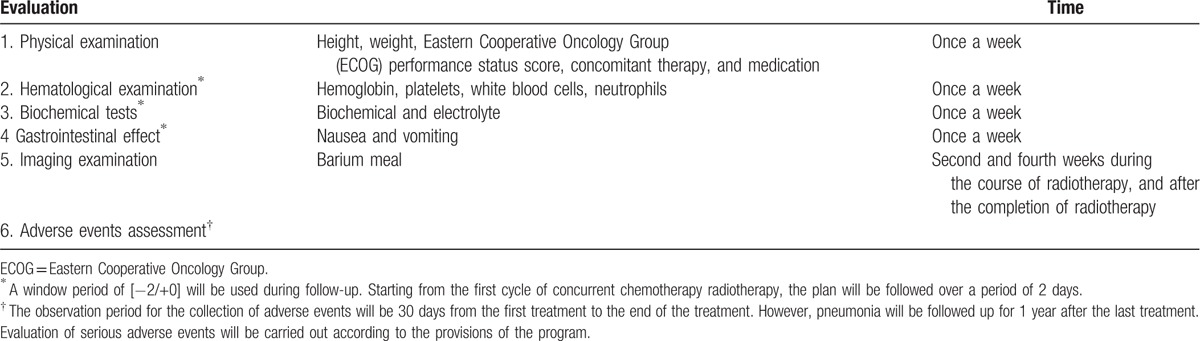
Evaluation of the treatment process.

The following conditions will be recorded at the end of the treatment (every 3 months during year 1–2, every 6 months thereafter):

1. Survival
2. Physical condition based on the ECOG performance status score
3. A physical examination
4. Vital signs
5. Electrocardiogram
6. A barium meal
7. Esophageal endoscopic biopsy (at the first follow-up time, and then at least once a year)
8. CT scan for tumor evaluation (at the first follow-up time, and then at least once every 6 months).

### 3.6 Statistical analysis and randomization

At a bilateral test 0.05 significance level and a power of 85%, with a dropout rate for each group of 10%, observe 40% endoscopic CR, and to exclude a lower limit of confidence interval (CI) of 20% CR, the study requires a total of 88 samples. Randomization will be administered centrally by the SPSS. Patients who meet the inclusion criteria will be randomized on a 1:1 basis to one of the 2 groups.

The proportion of patients with a clinical response and their 95% confidence intervals will be calculated and reported. The number and proportion of specific cases will be analyzed. The Chi-square test and Kaplan–Meier method will be used to analyze the rates and severity of disease progression and overall survival rate, respectively, according to NCI-CTCAE (version 4), and a Fisher exact (probability) test will be used to analyze the correlations between clinical outcomes and toxicity.

### 3.7 Ethics

The trial received ethical approval from the Ethics Committee of Mianyang Central Hospital, Sichuan, China (number: S2016055). The trial is subject to the supervision and management of the ethics committee.

### 3.8 Trial status

This study opened to recruitment in October 2016, with a planned recruitment period of 1.5 years.

## 4 Discussion

CRT has been accepted nowadays as the standard nonsurgical treatment for locally advanced EC.^[24]^ For 2 decades, chemotherapy, especially the 2-drug combination of cisplatin plus fluorouracil has been regarded as a standard regimen to treat patients with EC with distant metastases or recurrence.^[25]^ However, some retrospective studies have suggested that for patients with EC receiving radiotherapy combined with 2-drug chemotherapy have the severe toxicity.^[7,13–17]^ Our retrospective analysis of 133 cases of esophageal squamous cell carcinoma with complete statistical data found that for both OS and PFS, there was no significant difference between single and 2-drug chemotherapy combined with synchronous radiotherapy (unpublished). So we design this experiment to compare the complete remission rate of S-1 and S-1 combined with cisplatin in the treatment of esophageal squamous cell carcinoma.

*Expectations*: Compare S-1 plus cisplatin concurrent intensity-modulated radiotherapy for patients with esophageal squamous cell carcinoma, S-1 concurrent intensity-modulated radiotherapy has the same effect and less side effects.
